# SARS-CoV-2 receptor ACE2 is co-expressed with genes related to transmembrane serine proteases, viral entry, immunity and cellular stress

**DOI:** 10.1038/s41598-020-78402-2

**Published:** 2020-12-08

**Authors:** Wasco Wruck, James Adjaye

**Affiliations:** Institute for Stem Cell Research and Regenerative Medicine, Medical Faculty, Heinrich Heine University, Moorenstr.5, 40225 Düsseldorf, Germany

**Keywords:** Mechanisms of disease, Computational biology and bioinformatics

## Abstract

The COVID-19 pandemic resulting from the severe acute respiratory syndrome coronavirus 2 (SARS-CoV-2) which emerged in December 2019 in Wuhan in China has placed immense burden on national economies and global health. At present neither vaccination nor therapies are available. Here, we performed a meta-analysis of RNA-sequencing data from three studies employing human lung epithelial cells. Of these one focused on lung epithelial cells infected with SARS-CoV-2. We aimed at identifying genes co-expressed with angiotensin I converting enzyme 2 (ACE2) the human cell entry receptor of SARS-CoV-2, and unveiled several genes correlated or inversely correlated with high significance, among the most significant of these was the transmembrane serine protease 4 (TMPRSS4). Serine proteases are known to be involved in the infection process by priming the virus spike protein. Pathway analysis revealed virus infection amongst the most significantly correlated pathways. Gene Ontologies revealed regulation of viral life cycle, immune responses, pro-inflammatory responses- several interleukins such as IL6, IL1, IL20 and IL33, IFI16 regulating the interferon response to a virus, chemo-attraction of macrophages, and cellular stress resulting from activated Reactive Oxygen Species. We believe that this dataset will aid in a better understanding of the molecular mechanism(s) underlying COVID-19.

## Introduction

Severe acute respiratory disease COVID-19 is a result of infections with the coronavirus SARS-CoV-2 first reported in the Chinese city Wuhan, Province Hubei, in December 2019 and has since 11 March 2020 been designated as a pandemic by WHO. The origin of the virus is most likely zoonotic^1,2^ but the exact species transferring it is still under investigation as some studies suggest that it was transferred from pangolins^3^ or bats^4^. Tilocca et al*.* analysed the SARS-CoV-2 nucleocapsid and envelop proteins and found besides highest similarities with bat and pangolin also considerable similarities with dog, cat, cattle and other species^5,6^. Based on this, they suggest that earlier contact to similar viruses hosted by other species might be responsible for protection or—in case of multiple contacts—for antibody defense enhancement^7^. At the end of April 2020, the number of globally confirmed cases of COVID-19 exceeded 3 million and recorded deaths beyond 200,000 in the real-time statistics of the Johns Hopkins University^8^. Due to many unreported and asymptomatic cases, the infection fatality rate (IFR) is difficult to determine however Verity et al*.* estimate approximately 0.66% (0·39–1·33) in China^9^. The age-associated IFR is approximately 7.8% for those above 80 years^9^. Drugs have been re-purposed for stabilizing COVID-19, but these are not effective therapies^10^, examples include hydroxy-chloroquine (Malaria)^11,12^ and nelfinavir (HIV)^13^. However, remdesivir which was designed for Ebola treatment^11,12^ was at least shown to shorten the time to recovery and to reduce infection in the lower respiratory tract^14^. Another treatment option is to indirectly immunize individuals with plasma from convalescent COVID-19 patients^15^. Further approaches aim at mimicking the human virus cell entry receptor ACE2^4^ with human recombinant soluble ACE2 (hrsACE2)^16^. The cell entry receptor ACE2 associates with transmembrane proteases which prime the spike protein of the virus. Hoffmann et al*.* assigned this task to the protein TMPRSS2^17^ which they identified in the predecessor virus SARS-CoV and showed it is the same for SARS-CoV-2. The protease can be inhibited by existing compounds to interrupt further propagation of the virus in the human host. Several other publications confirmed the role of TMPRSS2 including a study by Matsuyama et al*.* showing enhanced SARS-CoV-2 infection in TMPRSS2 expressing VeroE6 cells^18^ and a study by Sungnak et al*.* reporting co-expression of ACE2 and TMPRSS2 in multiple tissues^19^. Collin et al*.* found that SARS-CoV-2 may enter the human body through the ocular surface epithelium mediated by ACE2 and TMPRSS2^20^.

Interestingly, ACE2 has been reported to be down-regulated following lung injury by Imai et al*.*^21^ and in the previous virus SAR-CoV by Haga et al*.*^22^ via a mechanism involving TNF-α-converting enzyme (TACE) shedding of the ectodomain of ACE2.

Here, we describe a meta-analysis focussing on the transcriptome data from human lung epithelial cells including samples infected with SARS-CoV-2 from a study described by Blanco Melo et al*.*^23^. We directed the exploration to co-expression with the known SARS-CoV-2 receptor ACE2. The analysis revealed a signature consisting of 72 genes significantly co-expressed with ACE2 either with positive or negative Pearson correlation. Of the transmembrane serine proteases, the most significantly co-expressed with ACE2 was TMPRSS4, suggesting it to be a putative druggable target.

## Results

### Cluster analysis of SARS-CoV-2 infected cells compared to other non-infected lung cells

**Figure 1:**
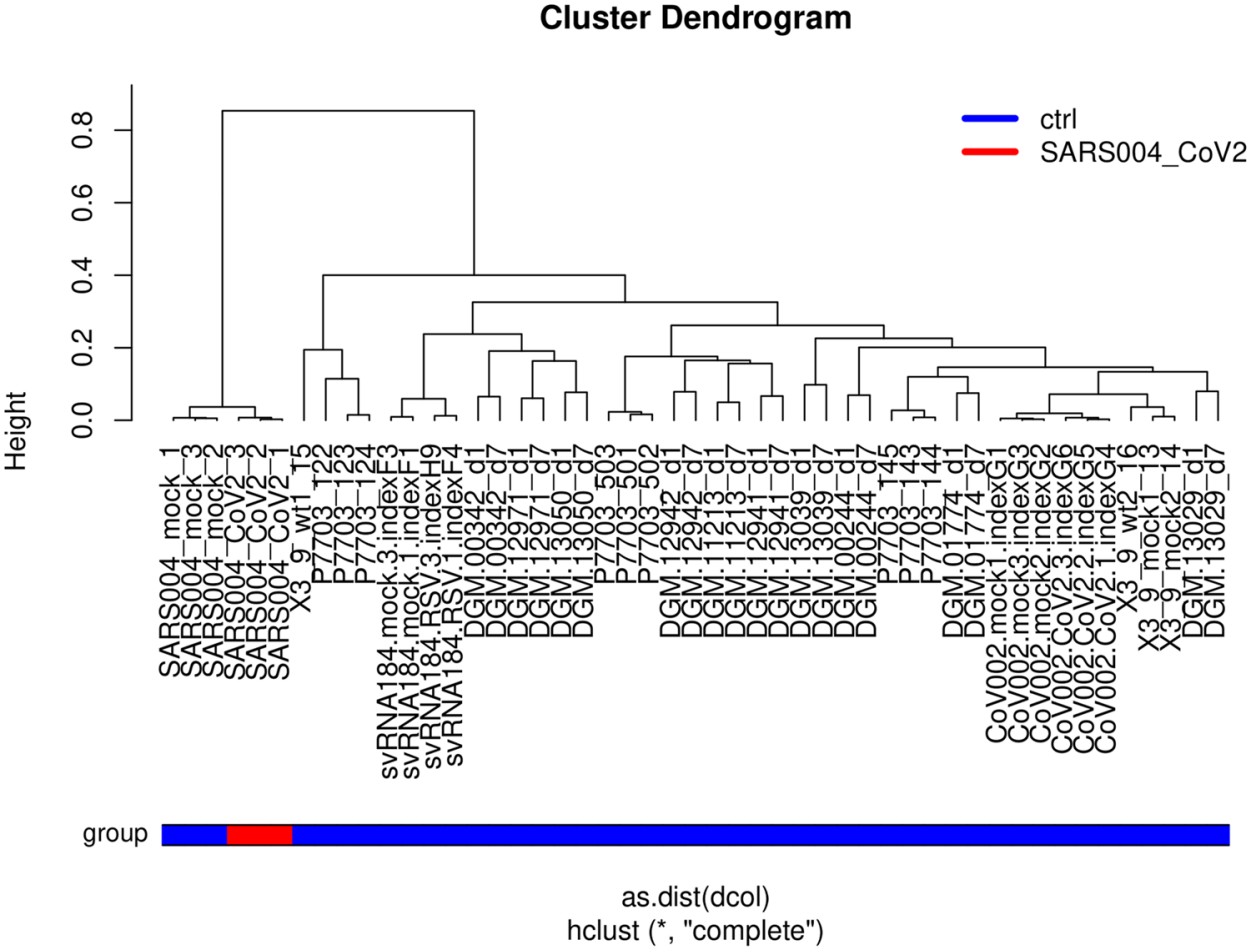
Lung epithelial cells (labeled “SARS004”) infected with SARS-CoV-2 cluster together with mock-infected lung epithelial cells but separated from all other lung epithelial cells.

Figure 1 shows a hierarchical cluster analysis of all samples used in this meta-analysis. Lung epithelial cells (labeled “SARS004” in Fig. 1) infected with SARS-CoV-2 cluster together with mock-infected lung epithelial cells but separated from all other lung epithelial cells and lung carcinoma cell lines which together we consider as control in this analysis. The table of Pearson correlation co-efficients (suppl. Table 2) reflects the grouping implicated by the hierarchical clustering: SARS-CoV-2 samples have highest correlation (r = 0.9884–0.9936) to the mock-infected SARS cells (Table 1).

**Table 1:** Datasets used in this focussed meta-analysis.

Dataset	Description	Reference
GSE147507	Primary human bronchial epithelial cells and lung adenocarcinoma infected with SARS-CoV-2 or Mock	Blanco-Melo, D. et al. Cell 181, 1036–1045.e9 (2020)
GSE146482	Human bronchial epithelium cell line BEAS-2B	Mukherjee SP et al. unpublished
GSE85121	Small airway epithelium brushing	Staudt MR et al. Respir Res 2018 May 14;19(1):78. PMID: 29,754,582

### Analysis of genes with correlated and anti-correlated expression with ACE2

**Figure 2:**
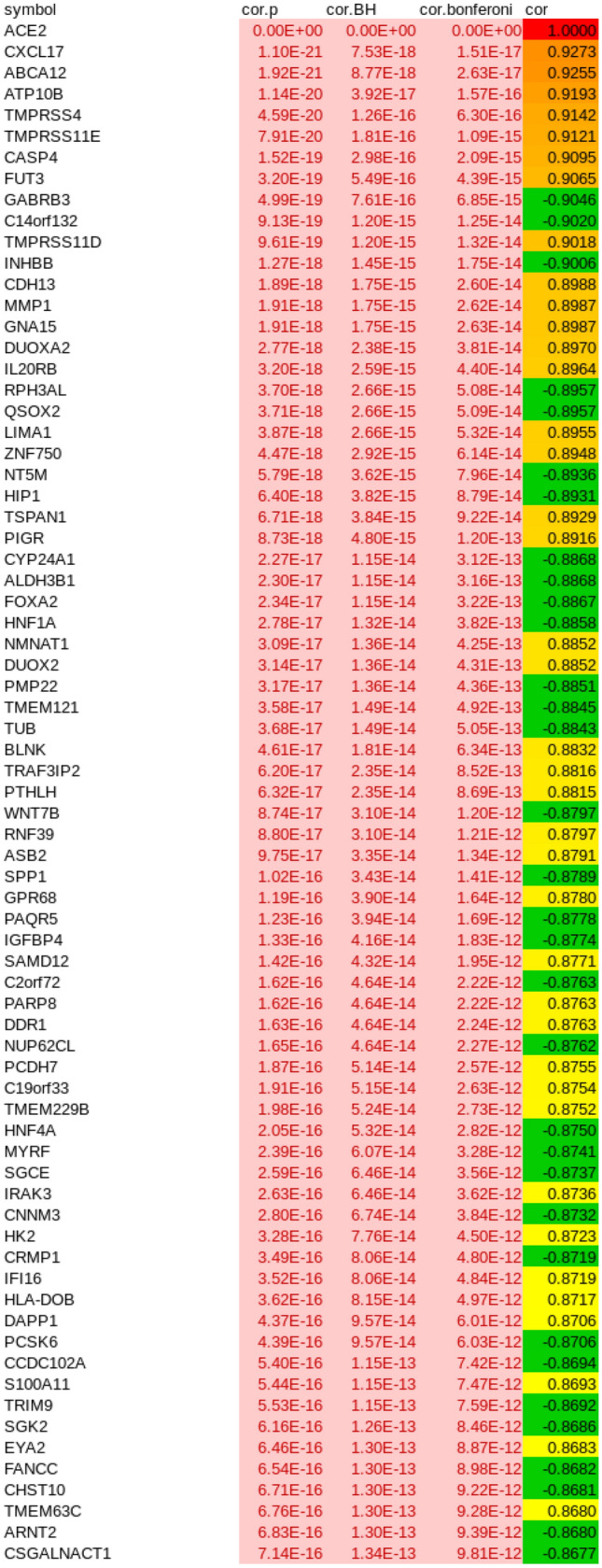
Most significantly (Bonferoni-adjusted p < 1E−11) correlated (red to yellow in last column) or anti-correlated (green) genes with ACE2. The transmembrane serine protease 4 (TMPRSS4) is one of the most significantly correlated (r = 0.9142, p = 4.59E−20) with ACE2 suggesting a major role in priming the SARS-CoV2 spike protein. CXCL17 (r = 0.9273, p = 1.1E−21), ABCA12 (r = 0.9256, p = 1−92E−21) and ATP10B (r = 0.9193, p = 1.14E−20) had marginally higher correlation with ACE2 while another transmembrane protease TMPRS11E (r = 0.9121, p = 7.91E−20) had slightly lower correlation.

**Figure 3:**
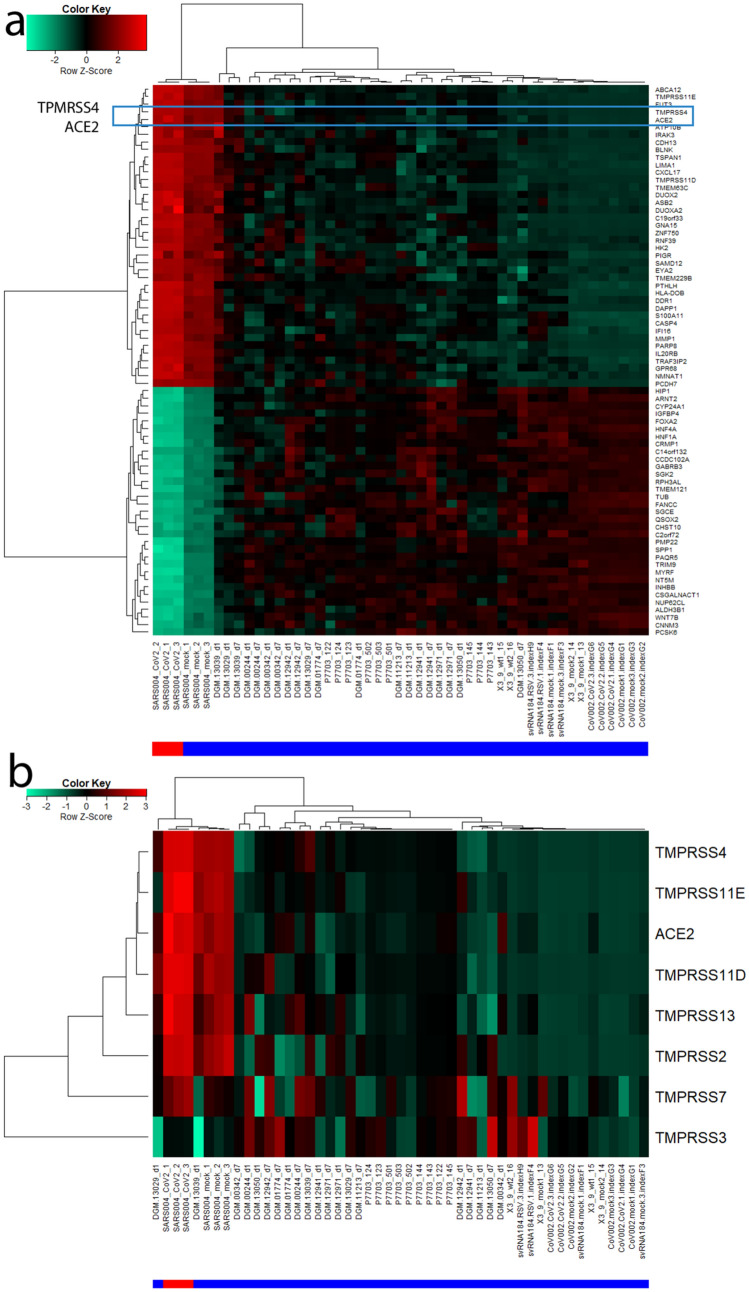
(**a**) Cluster analysis and gene expression heatmap of 72 most significantly (Bonferoni-adjusted p < 1E−11) correlated and anti.correlated genes with ACE2 shows close clustering of the serine protease TMPRSS4 with ACE2. (**b**) Heatmap of TMPRSS family members shows close clustering of TMPRSS11D/E and TMPRSS4 with ACE2 but also TMPRSS2 and TMPRSS13 have similar expression, particularly in SARS-CoV-2 infected samples. The red color bar indicates SARS-CoV-2, blue color bar control.

Building on the knowledge about ACE2 as receptor of the SARS-CoV-2 virus we set out to indentify genes with highly correlated expression with ACE2 with the aim of elucidating the molecular mechanisms underlying COVID-19. Figure 2 shows the genes most significantly (Bonferoni-adjusted p < 1E−1) correlated (red to yellow in last column) or anti-correlated (green) with ACE2 (full table in suppl. Table 3). The transmembrane serine protease 4 (TMPRSS4) is one of the most significantly correlated (r = 0.9142, p = 4.59E−20) with ACE2 therefore implying a major role in priming the SARS-CoV-2 spike protein. CXCL17 (r = 0.9273, p = 1.1E−21), ABCA12 (r = 0.9256, p = 1-92E−21) and ATP10B (r = 0.9193, p = 1.14E−20) had marginally higher correlation with ACE2 while another transmembrane protease TMPRS11E (r = 0.9121, p = 7.91E-20) had a slightly lower correlation. The expression of CXCL17 is probably due to a reaction to the infection by chemo-attracting macrophages^24^. The role of the ATP binding cassette subfamily A member 12 (ABCA12) is not fully elucidated with respect to COVID-19 but assumed to transport lipid via lipid granules to the intracellular space and transporting specific proteases – in the case of harlequin ichtyosis associated with desquamation^25^. Current knowledge on ATP10B is scarce. However, Wilk et al*.* (Table 3 in their publication) report Atp10b gene expression levels as highly inversely (negative) correlated with influenza gene expression changes in infected C57BL/6 J mice^26^. In Fig. 3a a cluster analysis and gene expression heatmap of the 72 most significantly (Bonferoni-adjusted p < 1E−11) correlated and anti-correlated genes with ACE2 shows close clustering of the serine protease TMPRSS4 with ACE2. Also in this analysis of 72 genes, SARS-CoV-2 (red color bar) cluster together with mock-infected SARS lung epithelial cells and separated from the other lung cells (blue color bar indicates control). In the heatmap presented in Fig. 3b, TMPRSS family members TMPRSS11D/E and TMPRSS4 show close clustering with ACE2 but also TMPRSS2 and TMPRSS13 have similar expression in all experiments, especially in the SARS-CoV-2 infected samples.

### Pathway analysis of genes co-regulated with ACE2

**Figure 4:**
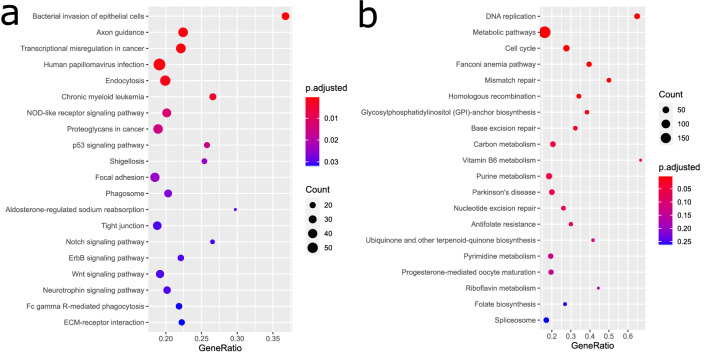
(**a**) The five most significantly overrepresented pathways correlated with ACE2 are *Human papillomavirus infection, Bacterial invasion of epithelial cells, Endocytosis, Axon Guidance* and *Transcriptional mis-regulation in cancer*. (**b**) The six most significantly overrepresented pathways anti-correlated with ACE2 are *DNA replication, Metabolic pathways, Cell cycle, Fanconi anemia pathway, Mismatch repair* and *Homologous recombination*. Many of these pathways are associated with DNA processing or repair. That these are down-regulated upon infection with SARS-CoV-2 is in line with reports about interferon and interferon stimulated genes (ISGs) inhibiting virus replication^16^. This would be a defense against the attempts of the virus to recruit the host’s DNA repair and homologous recombination mechanisms as Gillespie et al. report^29^.

In order to investigate the functionality of genes interacting with *ACE2* we filtered genes correlated with ACE2 by Bonferoni-adjusted p value < 0.05 and Pearson correlation coefficient > 0.6. 1891 genes fulfilled these criteria and were subjected to over-representation analysis of KEGG pathways^27^. The most significantly over-represented pathways associated with the 1891 genes correlated with *ACE2* (Fig. 4a, suppl. Table 4) are for example, *Bacterial invasion of epithelial cells (q* = *4.4E−06), Human papillomavirus infection (q* = *0.0006), Transcriptional misregulation in cancer (q* = *0.0006)* and *Endocytosis (q* = *0.002)*. This reflects the mechanisms of virus infection via invasion of epithelial cells and endocytosis.

### Pathway analysis of genes anti-correlated with ACE2

Analogously to the positively correlated genes we also examined the negatively correlated genes with ACE2 by filtering for Bonferoni-adjusted p value < 0.05 and Pearson correlation coefficient < − 0.6. 1993 genes passed these filtering criteria and were subjected to over-representation analysis of KEGG pathways^27^. The most significantly over-represented pathways in the 1993 genes negatively correlated with *ACE2* (Fig. 4b, suppl. Table 5) are *DNA replication (q* = *1E−12), Metabolic pathways (q* = *1.86E.08), Cell cycle (q* = *1.1E−05), Fanconi anemia pathway (q* = *1.24E−05), Mismatch repair (q* = *9.89E−05)* and *Homologouos recombination (q* = *0.0046)*. Many of these pathways are associated with DNA processing or repair. That these are over-represented in genes down-regulated upon infection with SARS-CoV-2 is in line with reports about interferon and interferon stimulated genes (ISGs) inhibiting virus replication^28^. This would be a defense against the attempts of the virus to recruit the host’s DNA repair and homologous recombination mechanisms^29^.

### GO analysis of genes co-regulated with ACE2

**Figure 5:**
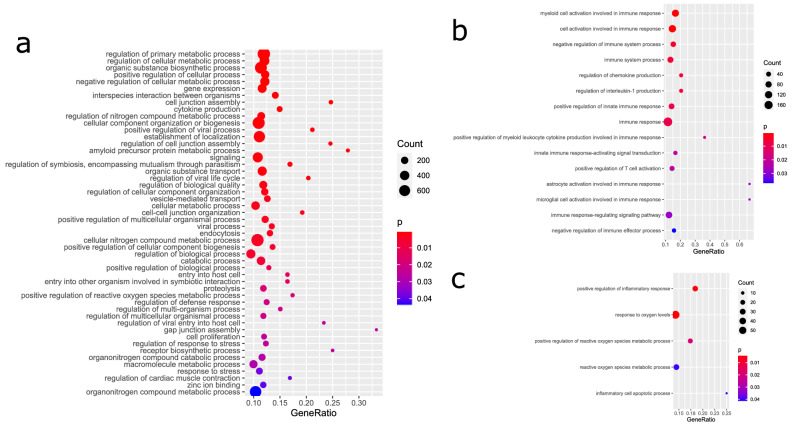
GO analysis reflects virus entry and immune response involving ROS and inflammation. (**a**) Selected GOs (Biological processes) shows interaction of virus and host, cell-junction organization, endocytosis, reaction involving cytokine production. (**b**) Immunity-related GOs illustrate the immune response involving activation of myeloid cells and T-cells and interleukin-1 and other chemokine production. (**c**) GOs associated with ROS and inflammation demonstrate involvement of ROS and inflammation leading to apoptosis.

We furthermore assessed the GOs over-represented in the 1891 genes positively and the 1993 genes negatively correlated with *ACE2*. Table 2 shows a selection of significant GOs from all three categories Biological Process (BP, Fig. 5a), Cellular Component (CC) and Molecular Function (MF), suppl. Table 6 provides the full table and suppl. Table 7 provides the full table for the 1993 genes negatively correlated with *ACE2*. Amongst the GO-BPs, metabolic processes are the most significant. GO-BP terms such as *Interspecies interaction between organisms, Cytokine production* and *positive regulation of viral process* reflect activated mechanisms post-viral infection. Interestingly, we found *regulation of coagulation* amongst the GO-BPs what may help elucidate reports about co-agulation in acro-ischemic COVID-19 patients^30^. In the GO-CCs, the terms *intracellular* and *membrane-bounded organelle* are most significant. In the GO-MFs, the terms *metal ion binding* and *protein binding* emerge as most significant due probably reflecting the binding of the virus proteins to the host cells. For the full gene lists associated with these terms refer to suppl. Tables 5 and 6.

**Table 2:** Selected over-represented GOs in genes significantly correlated with ACE2.

GO_BP term	P value	GO_CC term	P value	GO_MF term	P value
Regulation of primary metabolic process	5.23E−11	Intracellular	3.35E−27	Metal ion binding	4.98E−08
Regulation of cellular metabolic process	6.04E−09	Membrane−bounded organelle	8.95E−23	Protein binding	1.84E−05
Organic substance biosynthetic process	8.56E−07	Extracellular exosome	9.79E−18	Catalytic activity, acting on a protein	6.44E−03
Positive regulation of cellular process	2.09E−06	Extracellular organelle	1.21E−17	Metallopeptidase activity	4.99E−02
Negative regulation of cellular metabolic process	2.69E−05	Cytoplasm	2.07E−14		
Gene expression	3.24E−05	Vesicle	8.85E−14		
Interspecies interaction between organisms	3.30E−05	Ion binding	5.22E−08		
Cell junction assembly	1.20E−04	Membrane raft	2.03E−04		
Cytokine production	2.21E−04	Membrane region	5.95E−04		
Regulation of nitrogen compound metabolic process	3.90E−04	Extracellular region	1.33E−03		
Cellular component organization or biogenesis	4.77E−04	Plasma membrane	5.48E−03		
Positive regulation of viral process	4.86E−04	Whole membrane	8.94E−03		
Establishment of localization	6.03E−04				
Regulation of cell junction assembly	7.11E−04				
Amyloid precursor protein metabolic process	7.30E−04				
Signaling	7.61E−04				
Regulation of symbiosis, encompassing mutualism through parasitism	9.15E−04				
Organic substance transport	9.22E−04				
Regulation of viral life cycle	1.04E−03				
Regulation of biological quality	1.31E−03				
Regulation of cellular component organization	1.69E−03				
Vesicle-mediated transport	2.18E−03				
Cellular metabolic process	2.21E−03				
Cell–cell junction organization	2.53E−03				
Positive regulation of multicellular organismal process	2.74E−03				
Viral process	2.81E−03				
Endocytosis	3.08E−03				
Cellular nitrogen compound metabolic process	3.48E−03				
Positive regulation of cellular component biogenesis	5.04E−03				
Regulation of biological process	5.23E−03				
Catabolic process	6.34E−03				
Positive regulation of biological process	1.09E−02				
Entry into host cell	1.27E−02				
Entry into other organism involved in symbiotic interaction	1.27E−02				
Proteolysis	1.72E−02				
Positive regulation of reactive oxygen species metabolic process	1.85E−02				
Regulation of defense response	2.06E−02				
Regulation of multi-organism process	2.09E−02				
Regulation of multicellular organismal process	2.12E−02				
Regulation of viral entry into host cell	2.49E−02				
Gap junction assembly	2.51E−02				
Cell proliferation	2.52E−02				
Regulation of response to stress	2.57E−02				
Receptor biosynthetic process	2.68E−02				
Organonitrogen compound catabolic process	2.79E−02				
Macromolecule metabolic process	2.91E−02				
Response to stress	3.85E−02				
Regulation of cardiac muscle contraction	3.93E−02				
Zinc ion binding	4.07E−02				
Organonitrogen compound metabolic process	4.45E−02

### Immune system associated GOs co-regulated with ACE2

Table 3 and Fig. 5b show GOs (all Biological Processes) related to the immune system over-represented in genes correlated with *ACE2*. Myeloid cells involved in the immune response (GO:0,002,275, p = 5E−07) as well as T-cells (GO:0,050,870, p = 0.02) are activated. Chemokines, in particular interleukin-1 are produced (GO:0,032,642, p = 0.0049, GO:0,032,652, p = 0.0049) also IL33 and TNF. Additionally, positive regulation of the innate immune response was prominent (GO:0,045,089, p = 0.0079). *CXCL17* was the gene with the highest correlation with *ACE2* and is involved in immune system process—as described above by chemo-attracting macrophages^24^. Among the most significantly *ACE2*-correlated genes was *IFI16* which is associated with several immune system GOs and known as regulator of the interferon response to viruses^31^ and will be described in more detail in the next section about *Protein interaction networks*. Also in the protein-interaction network of the most significantly ACE2-correlated genes was the interleukin 20 receptor B (IL20RB) which appeared in several GOs listed in Table 3. With respect to viruses, there is meagre knowledge on IL20RB, however a study reported over-expression in the pneumonia causing avian influenza A H7N9 virus^32^.

**Table 3:** GOs (all biological process) related to immune system in genes correlated with *ACE2.*

Term	P value
Myeloid cell activation involved in immune response	5.3978E−07
Cell activation involved in immune response	4.3656E−05
Negative regulation of immune system process	0.00331363
Immune system process	0.0048604
Regulation of chemokine production	0.00486129
Regulation of interleukin-1 production	0.00486129
Positive regulation of innate immune response	0.0078574
Immune response	0.00855493
Positive regulation of myeloid leukocyte cytokine production involved in immune response	0.01809849
Innate immune response-activating signal transduction	0.02190672
Positive regulation of T cell activation	0.02209419
Astrocyte activation involved in immune response	0.02764374
Microglial cell activation involved in immune response	0.02764374
Immune response-regulating signaling pathway	0.0286071
Negative regulation of immune effector process	0.03757784

### GOs associated with inflammation and reactive oxygen species (ROS) amongst genes co-regulated with ACE2

Table 4 and Fig. 5c show GOs (all Biological Processes) related to inflammation and ROS over-represented in genes correlated with ACE2. The GO, *positive regulation of inflammatory response* (p = 0.0039) would imply that an inflammatory process is induced which finally leads to apoptosis—as demonstrated by the GO *inflammatory cell apoptotic process* (p = 0.042). The virus receptor ACE2 is involved in the *positive regulation of reactive oxygen species metabolic process* (p = 0.0185). Induction of ROS by a respiratory virus and subsequent inflammation has been reported Jamaluddin et al*.*^33^.

**Table 4:** GOs (all biological process) related to inflammation and reactive oxygen species (ROS) in genes correlated with *ACE2.*

Term	P value
Positive regulation of inflammatory response	0.0039263
Response to oxygen levels	0.00438988
Positive regulation of reactive oxygen species metabolic process	0.01851891
Reactive oxygen species metabolic process	0.04088524
Inflammatory cell apoptotic process	0.04206722

### Protein-interaction networks

**Figure 6:**
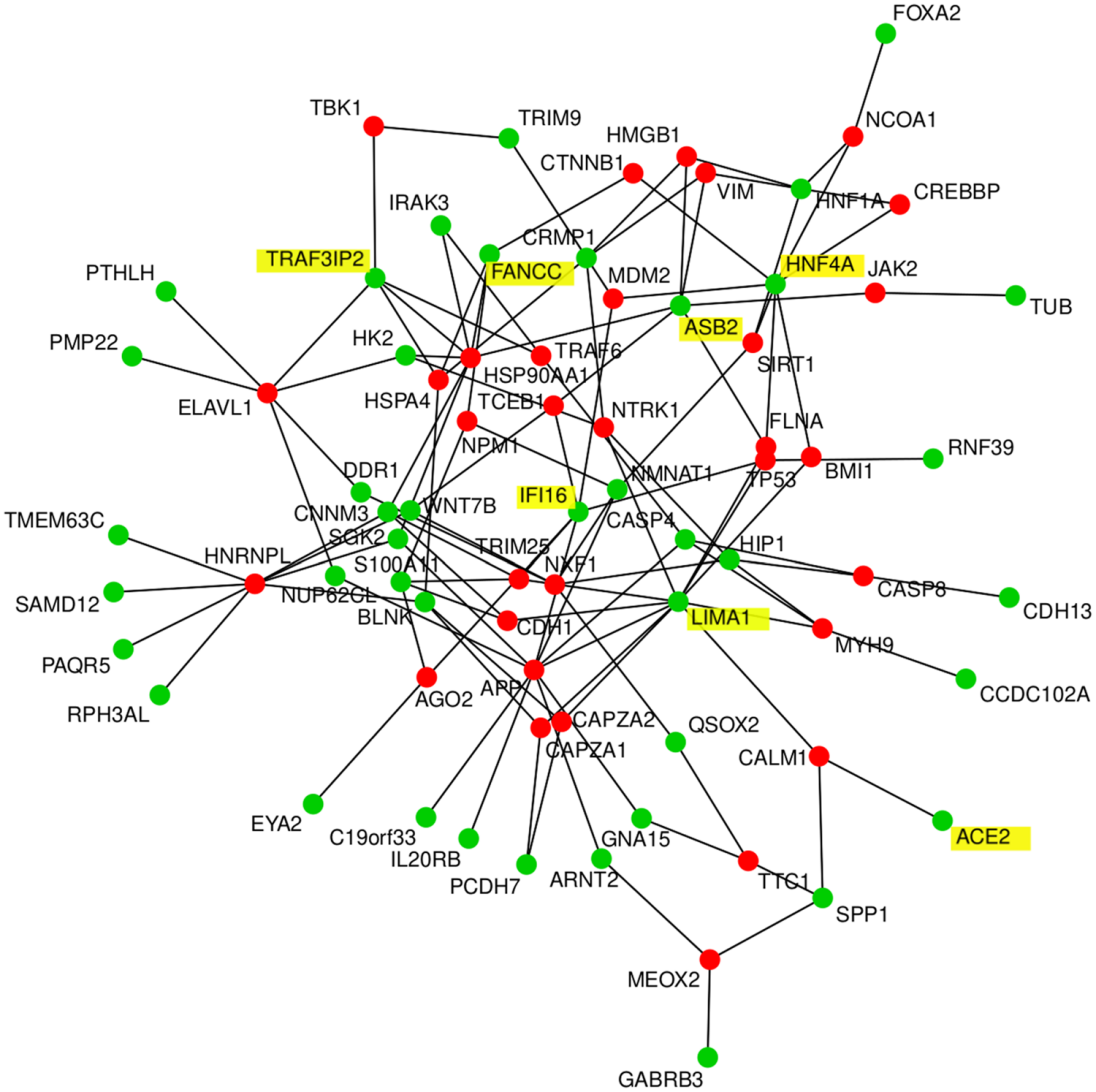
Protein interaction network of genes most significantly (Bonferoni-adjusted p < 1E−11) correlated and anti-correlated genes with ACE2 shows IFI16 (r = 0.8719), LIMA1 (r = 0.8955), CNNM3 (r = − 0.8732), HNF4A (r = − 0.8750), TRAF3IP2 (r = 0.8816), ASB2 (r = 0.8791) and FANCC (r = − 0.8682) as hub genes. Genes found as interactors in the BioGrid database are marked in red, the original geneset of *ACE2*-correlated genes is marked in green, hub genes and *ACE2* have yellow shading.

We further restricted the set of ACE2-correlated or -anti-correlated genes by drastically filtering with Bonferoni-adjusted p < 1E−11 in order to construct a human readable protein interaction network of the most significant proteins (Fig. 6). The protein-interaction network generated from correlated and anti-correlated genes with *ACE2* shows *IFI16 (r* = *0.8719), LIMA1 (r* = *0.8955), CNNM3 (r* = *− 0.8732), HNF4A (r* = *− 0.8750), TRAF3IP2 (r* = *0.8816), ASB2 (r* = *0.8791)* and *FANCC* (r = − 0.8682) as hub genes (interactors from the BioGrid database are marked in red, original *ACE2*-correlated/anti-correlated genes are marked in green, hub genes and *ACE2* are highlighted with yellow shading ). Interferon plays a major role in the host response to a virus and Thompson et al*.* reported that IFI16 – one of the hub genes in our network—controls the interferon response to DNA and RNA viruses^31^. Lin and Richardson review that *LIMA1* (formerly EPLIN) – which is known to enhance bundling of actin filaments^34^—mediates the interaction between Cadherins and Actin in the context of adherens junctions—playing a role in measles virus infection—via trans-binding with molecular interactors on adjacent cells^35^. In line with this, LIMA1 is connected with E-cadherin (CDH1) in the network and also Cadherin 13 (CDH13) is part of it and among the most significantly correlated genes to ACE2. The connection of ACE2 to calmodulin 1 (CALM1) is based on a publication by Lambert et al*.*^36^ in which they show that CALM1 interacts with the corona virus receptor ACE2 and inhibits shedding of its ectodomain^36^. CALM1 inhibitors in turn can reverse this process so that the ACE2 ectodomain is shed, and is partially mediated by a metalloproteinase^36^. The direct connection between CALM1 and LIMA1 was found in a large-scale interactome study^37^. The involvement of the hub gene Fanconi anemia complementation group C (FANCC), although not experimentally proven, might reflect recruitment of DNA repair and homologous recombination mechanisms from the host by the virus.

### Role of TMPRSS4 and other TMPRSS gene family members

A pivotal result of this meta-analysis is the transmembrane serine protease TMPRSS4 which was one of the most significantly correlated genes with ACE2. Additionally TMPRSS11E (r = 0.9121, Bonferoni-corrected p = 1.09E−15) and TMPRS11D (r = 0.9018, Bonferoni-corrected p = 1.32E−14) from the same gene family were found more significant than TMPRSS2 which however was still significantly correlated with ACE2 (r = 0.767, Bonferoni-corrected p = 1.79E−6). The assignment of the SARS-CoV-2 spike protein priming functionality to TMPRSS2 was based on the assumption that it would be the same as for its predecessor SARS-CoV^17^ and was confirmed by several publications^19,20^. However, besides TMPRSS2 other proteases might be involved—see the heatmap in Fig. 3b which illustrates expression of transmembrane serine proteases. The highly significant co-expression of TMPRSS4 with ACE2 and its relevant role as transmembrane serine protease has enabled us to hypothesize that TMPRSS4 might also be involved in priming the SARS-CoV-2 spike protein. We therefore anticipate that inhibitors of TMPRSS4, TMPRS11D and TMPRS11E—besides those for TMPRSS2—could be a promising subject of further research.

### Validation with a dataset of SARS-CoV-2 infected human bronchial organoids

We validated our results with the RNAseq dataset GSE150819 of human bronchial organoids infected with SARS-CoV-2^38^. Suppl. Table 8 shows the correlation of members of the TMPRSS gene family with ACE2 in this dataset. As in the first analysis of lung epithelial cells (Fig. 2) TMPRSS4 is also the gene most significantly correlated with ACE2 expression (p = 4.45E−05, Benjamini-Hochberg-corrected p = 0.0006, r = 0.86 ). Also TMPRSS2 (p = 0.014379, Benjamini-Hochberg-corrected p = 0.042562, r = 0.62 ) and TMPRSS11D (p = 0.000456, Benjamini-Hochberg-corrected p = 0.003185, r = 0.79 ) have significant p values and also Benjamini-Hochberg-corrected p values while TMPRSS11E here is not significant.

## Discussion

In this meta-analysis we compared RNA-seq data of lung cells infected with SARS-CoV-2 and other lung cells with particular focus on correlated gene expression with the known SARS-CoV-2 receptor gene *ACE2*. We identified a signature of genes positively or negatively correlated with *ACE2* amongst which the most outstanding was the transmembrane serine protease *TMPRSS4*. In a recent publication Hoffmann et al.^17^. Inferred from the knowledge that the preceeding virus, SARS-CoV, uses ACE2 as receptor for entry and the serine protease TMPRSS2 for spike protein priming that the new virus SARS-CoV-2^17^ would do the same. While the involvement of the receptor ACE2 appears to be established^3,16^ the use of TMPRSS2 for spike protein priming appears not fully settled as Hoffmann et al*.* still have to concede “that SARS-CoV-2 infection of Calu-3 cells was inhibited but not abrogated by camostat mesylate” (serine protease inhibitor with activity against TMPRSS2)^17^. The high significance (r = 0.9142, p = 4.59E−20) in our co-expression analysis with ACE2 suggests that TMPRSS4 is a considerable candidate for spike protein priming. That is in line with findings by Zang et al*.* who reported that TMPRSS4 besides TMPRSS2 enhances infection of small intestinal enterocytes with SARS-CoV-2^39^. However, TMPRSS4 is closely related to TMPRSS2 which both can proteolytically cleave the hemagglutinin of influenza viruses^40^. Further transmembrane serine proteases TMPRS11D (r = 0.9018, p = 9.61E−19) and TMPRS11E (r = 0.9121, p = 7.91E−20) in our analysis also emerged more significant than TMPRSS2 (r = 0.767, p = 1.3E−10). The TMPRS11 family member TMPRS11A was found to enhance viral infection with the first coronavirus SARS-CoV by spike protein cleavage in the airway^41^. Thus, we should not exclude the probability that other members of the TMPRSS gene family may be proteases for the SARS-CoV-2 spike protein. TMPRSS2 inhibitors have been proposed by Hoffmann et al*.*^17^—and earlier for the SARS-Cov virus by Kawase et al*.*^42^ as working best together with cathepsin B/L inhibitors. We propose to investigate the effect of TMPRSS4 inhibitors in further research. As TMPRSS4 has been implicated in the invasion and metastasis of several cancers it has also been considered as target for cancer therapy for which a modest inhibitory effect of the above mentioned inhibitors in TMPRSS4-overexpressing SW480 cells was reported^43^. Interestingly, also *TMPRSS2* is connected with epithelial carcinogenesis as consistently over-expressed in prostate cancer^44^, and later a gene fusion of *TMPRSS2* and *ERG* was reported as the predominant molecular subtype of prostate cancer^45^, where *TMPRSS2* however only contributes untranslated sequence^46^. Assuming that co-expression with *ACE2* is an indication that TMPRSS4 may prime the SARS-CoV-2 spike protein we suggest that further development and testing of more effective TMPRSS4 inhibitors in in vitro and in vivo models could support the translation into clinical settings. However, we have to state the limitation that this study is a meta-analysis based solely on transcriptome and not proteome data.

Besides the identification of *TMPRSS4,* we found several significantly over-represented GOs and pathways such as Endocytosis, Papilloma virus infection and Bacterial invasion of epithelial cells for which we provide full gene lists to foster further elucidation of disease mechanisms. Genes from the constructed protein-interaction network provide a first snapshot of a comprehensive image: *IFI16* controls the interferon response to the virus^31^, *LIMA1* mediating the interaction between Cadherins (*CDH1*, *CDH13*) and Actin in the context of adherens junctions potentially playing a role in virus infection, *CALM1* inhibits shedding of the ectodomain of the virus receptor *ACE2*^36^.

**Figure 7:**
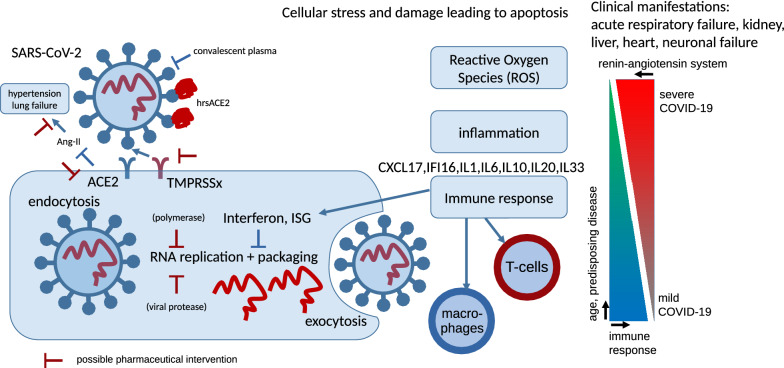
Scheme of SARS-CoV-2 infection. The coronavirus SARS-CoV-2 docks at the receptor ACE2 on the membrane of the human epithelial cell. Transmembrane serine proteases TMPRSSx mediate SARS-CoV-2 cell entry via ACE2. TMPRSS2 was reported for this in the first SARS-CoV and by previous publication also for SARS-CoV-2 but we hypothesize that due to co-expression with ACE2, TMPRSS4 and other members of the TMPRSS family may well perform this task. We suggest that inhibitors of TMPRSS4 and other TMPRSS family members might have therapeutic potential. Upon entry into the cell, viral RNA is released, replicated and packaged again. Replication can be inhibited by interferon and interferon stimulated genes (ISG) what we also saw in negatively correlated replication pathways (e.g. DNA replication and homologous recombination). This indicates a healthy immune response and may be impaired in persons with a weak immune system due to age or disease. The packaged virus is released from the cell and can be attacked by macrophages chemo-attracted by CXCL17—or T-cells for which we found evidence for by GO analysis and by associated interleukins IL1 and IL7. It is tempting to speculate that the severity of the clinical manifestations such as the acute respiratory failure and also failure in other organs depends on the quality of the immune system decreasing with age or diseases such as diabetes. The involvement of ACE2 in the renin-angiotensin system as antagonist of ACE in regulating blood pressure via Angiotensin II (Ang-II), vasoconstriction, dilation and its protective role against lung injury are additional factors which correlate with age^21,50,53^.

Furthermore, GO analyses revealed several biological processes related to viral cell entry, host reaction, immune response, ROS, inflammation and apoptosis. This led us to propose a cascade of events taking place post SARS-CoV-2 entry into host cells- illustrated in Fig. 7 together with possible drug targets. The coronavirus SARS-CoV-2 docks at the receptor ACE2 on the membrane of the human epithelial cell, the early stage of infection. According to reports by Monteil et al*.* these processes can be blocked with recombinant hrsACE2^16^. Transmembrane serine proteases TMPRSS mediate SARS-CoV-2 cell entry via ACE2^17,47^. TMPRSS2 has been described as a mediator of ACE2-coupled endocytosis in the first SARS-CoV^48^ and by a previous publication also for SARS-CoV-2^17^. However, we identified high levels of co-expression between ACE2 and TMPRSS4 and other members of the TMPRSS family and hypothesize that any of these additional family members might have the same function as the well described TMPRSS2. As a consequence, we propose that besides TMPRSS2 also other TMPRSS family members could be targets of pharmaceutical intervention warranting further research. After entering the cell, SARS-CoV-2 RNA is released, replicated and packaged again. Drugs can target the viral protease and the polymerase needed for replication^49^. Replication can further be inhibited by interferon and interferon-stimulated genes (ISG)^28^ which we also found evidence for in negatively correlated replication pathways (e.g. DNA replication and homologous recombination). This depends on a healthy immune response and may be impaired in individuals with a weak immune system due to age or underlying diseases. The virus is packaged and released into the extracellular space where it can be attacked by macrophages chemo-attracted by CXCL17^24^. Also T-Cells can be involved in the immune response. We found evidence for their activation in GO analysis by associated interleukins IL1 and IL7. Although immunity is not the main focus of this manuscript it is tempting to speculate that the severity of the clinical manifestations such as the acute respiratory failure and also failure in other organs depend on the state of the immune system which decreases with age or diseases such as diabetes.The involvement of ACE2 in the renin-angiotensin system as antagonist of ACE in regulating blood pressure via Angiotensin II, vasoconstriction, dilation and its protective role against lung injury^41^ are additional factors which correlate with age^50^. This is confirmed by reports from Wadmann et al*.*^51^ about Centers for Disease Control and Prevention (CDC) data from 14 U.S. states that 50% hospitalized COVID-19 patients had pre-existing high blood pressure^51^. In their study about ACE2 in the preceeding SARS-CoV virus Imai et al*.*^21^ found that *ACE2* protects against lung injury and acid-induced lung injury in a *Ace2*-knockout mouse can be improved by an inhibitor of the Angiotensin II receptor AT1^21^. The results of clinical studies but also statistics on hypertension and even more important statistics on pharmacological treatment of hypertension in COVID-19 patients may shed light on the discussions if treatment with ACE inhibitors and angiotensin receptor blockers (ARBs) are detrimental^52^ or beneficial^53^.

We conclude, that our meta-analysis of RNA-Seq data of lung cells partially infected with SARS-CoV-2 identified the transmembrane serine protease *TMPRSS4* as one of the most significantly correlated genes with the virus receptor *ACE2*. The importance of this finding is underlined by Zang et al. who simultaneously with our preprint publication reported that TMPRSS4 enhances SARS-CoV-2 infection of small intestinal enterocytes^39^. We propose that inhibitors of TMPRSS family members TMPRSS4, TMPRSS11D and TMPRSS11E besides TMPRSS2 are worthwhile testing in i*n vitro* and i*n vivo* studies. As clinicians, pathologists and scientists are struggling to get to grips with and understand the damage wrought by SARS-CoV-2 as it invades the body, we hope that our analyses and dataset will contribute to a better understanding of the molecular basis of COVID-19.

## Methods

### Sample collection of lung cell RNA-Seq data

Next-generation sequencing datasets measured in RNA-Seq experiments with lung cells (GSE147507: Illumina NextSeq 500; GSE146482: Illumina NovaSeq 6000; GSE85121: Illumina HiSeq 2500) were downloaded from NCBI GEO (Table 1, suppl. Table 4). These datasets were provided along with studies by Blanco-Melo et al*.*^23^ (GSE147507) and Staudt e*t al.*^54^ (GSE85121). A final publication related to the dataset GSE146482 is yet to materialize. From accession no. GSE85121 only small airway epithelium brushing cells were used but alveolar macrophages were excluded while from accession no. GSE147507 only human epithelial and adenocarcinoma lung cells were used but ferret cells and updates after March 24 were excluded and from accession no. GSE146482 only control epithelial cells were used but graphene oxide treated cells were excluded. After exclusion of the above mentioned datasets not fitting the target cell type, 49 samples remained useful.

### Data normalization and analysis

After the excluded samples had been filtered from the downloaded RNA-Seq data data was imported into the R/Bioconductor environment^55,56^. Read counts from accesion nos. GSE147507 and GSE146482 were converted to FPKM (fragments per kilobase of exon model per million reads mapped) using trancript lengths downloaded from ENSEMBL (version GRCh38, p13). Batch effects were removed with the package sva^57^ employing method ComBat^58^. Normalization was performed via the voom method^59^. Pearson correlation coefficients between samples were calculated with the R-builtin method *cor*. Dendrograms were drawn employing the dendextend package^60^ filtering genes for high coefficient of variation above the 75% quantile.

The validation dataset GSE150819 of human bronchial organoids infected with SARS-CoV-2^38^ downloaded from NCBI GEO was normalized with the voom method^59^ before calculating correlation of each gene to ACE2 gene expression.

### Detection of genes correlated with ACE2

The Pearson correlation of the normalized gene expression values for all samples was calculated between the gene *ACE2* and each other gene. The method cor.test was applied to calculate the p value for the t test for Pearson correlation. The p value was Bonferoni-corrected by division by the number of genes and additionally adjusted via the Benjamini–Hochberg method^61^. Correlated genes were filtered with a very restrictive criterion (Bonferoni-adjusted-p < 1E−11)—in order to get human readable numbers of genes for heatmap and network generation- and conventional criteria (r > 0.6, Bonferoni-adjuste-p < 0.05) for positively correlated genes and (r < 0.6, Bonferoni-adjusted-p < 0.05) for negatively correlated genes.

### Pathway and gene ontology (GO) analysis

The R package GOstats^62^ was employed for over-representation analysis of positively and negatively correlated genes with the SARS-CoV-2 receptor gene *ACE2*. KEGG pathway annotations which had been downloaded from the KEGG database^27^ in March 2018 were used for testing over-representation of the positively and negatively ACE2-correlated genes via the R-builtin hypergeometric test.

Dot plots showing the p value of the hypergeometric test, the ratio of significant genes to all genes in the pathway and the number of significant genes per pathway were plotted via the package *ggplot2*^63^.

### Protein-interaction networks

A human protein interaction network was constructed in a similar manner as we described in our previous publication^64^. However, here only direct interactors and no further interactors of interactors were extracted from the Biogrid database version 3.4.161^65^ using the restrictively filtered (Bonferoni-adjusted-p < 1E−1) genes significantly correlated or anti-correlated with ACE2 gene expression. The network was reduced to the n = 30 nodes with most interactions and was plotted via the R package *network*^66^ showing original genes in green and BioGrid-derived interactors in red.

## Supplementary information

Supplementary Information 1.

Supplementary Information 2.

Supplementary Information 3.

Supplementary Information 4.

Supplementary Information 5.

Supplementary Information 6.

Supplementary Information 7.

Supplementary Information 8.

Supplementary Information 9.

## Data Availability

No datasets were generated during the current study. The datasets used for the meta-analysis are available at the National Center for Biotechnology Information (NCBI) Gene expression Omnibus (GEO) accessions referred to in Table 1.
