# Supplementary material for: SARS-CoV-2 receptor ACE2 is co-expressed with genes related to transmembrane serine proteases, viral entry, immunity and cellular stress
Source: Sci Rep. 2020 Dec 8;10:21415. doi: 10.1038/s41598-020-78402-2 (PMC7723043; doi:10.1038/s41598-020-78402-2)
Supplement: Supplementary file 8 — Supplementary Information 8. [file 41598_2020_78402_MOESM8_ESM.docx]

**Supplementary Table 8: Pearson correlations and associated p-values of genes from the TMPRSS family with ACE2 in the dataset GSE150819 containing lung organoids infected with SARS-CoV-2.**

| symbol | cor.p | cor.BH | cor.bonferoni | cor |
| --- | --- | --- | --- | --- |
| TMPRSS4 | 4.45E-05 | 0.000609 | 0.926503 | 0.857106 |
| TMPRSS11A | 4.52E-05 | 0.000615 | 0.941194 | 0.856739 |
| TMPRSS11BNL | 0.000103 | 0.001084 | 2.13684 | 0.836168 |
| TMPRSS11D | 0.000456 | 0.003185 | 9.497711 | 0.790115 |
| TMPRSS13 | 0.001294 | 0.006847 | 26.92977 | 0.749553 |
| TMPRSS2 | 0.014379 | 0.042562 | 299.1709 | 0.616495 |
| TMPRSS3 | 0.017747 | 0.050072 | 369.252 | 0.601257 |
| TMPRSS4-AS1 | 0.018874 | 0.052359 | 392.6913 | 0.596659 |
| TMPRSS11GP | 0.057632 | 0.121022 | 1199.082 | 0.500116 |
| TMPRSS11B | 0.093085 | 0.175635 | 1936.731 | 0.449107 |
| TMPRSS6 | 0.140595 | 0.241017 | 2925.226 | -0.39908 |
| TMPRSS11E | 0.141265 | 0.241782 | 2939.152 | 0.398463 |
| TMPRSS12 | 0.407391 | 0.541955 | 8476.174 | 0.231043 |
| TMPRSS7 | 0.454917 | 0.588766 | 9465.01 | 0.208917 |
| TMPRSS11F | 0.506259 | 0.636758 | 10533.22 | 0.186266 |
| TMPRSS9 | 0.543996 | 0.670235 | 11318.38 | -0.17029 |
| TMPRSS5 | 0.815247 | 0.878507 | 16962.03 | -0.06599 |
