# Supplementary material for: SARS-CoV-2 receptor ACE2 is co-expressed with genes related to transmembrane serine proteases, viral entry, immunity and cellular stress
Source: Sci Rep. 2020 Dec 8;10:21415. doi: 10.1038/s41598-020-78402-2 (PMC7723043; doi:10.1038/s41598-020-78402-2)
Supplement: Supplementary file 9 — Supplementary Information 9. [file 41598_2020_78402_MOESM9_ESM.pdf]

**Supplementary Material for „SARS-CoV-2 receptor ACE2 is co-expressed with genes related to transmembrane serine proteases, viral entry, immunity and cellular stress“**

by Wasco Wruck[1] and James Adjaye[1,*]

[1]Institute for Stem Cell Research and Regenerative Medicine, Medical Faculty, Heinrich Heine University, Moorenstr.5, 40225, Düsseldorf, Germany

[*] corresponding author: James Adjaye (james.adjaye@med.uni-duesseldorf.de)

**Supplementary Table 1 (tableS1.xlsx): Detailed description of all samples used in this meta-analysis**

**Supplementary Table 2 (tableS2.xlsx): Pearson correlation coefficients of gene expression of all samples used in this meta-analysis**

**Supplementary Table 3 (tableS3.xlsx): Pearson correlation coefficients and associated p-values (additionally Benjamini-Hochberg and Bonferoni correction) of genes to expression of ACE2**

**Supplementary Table 4 (tableS4.xlsx): Over-represented KEGG pathways in genes positively correlated with *ACE2* gene expression**

**Supplementary Table 5 (tableS5.xlsx): Over-represented KEGG pathways in genes negatively correlated with *ACE2* gene expression**

**Supplementary Table 6 (tableS6.xlsx): Over-represented GOs in genes positively correlated with *ACE2* gene expression**

**Supplementary Table 7 (tableS7.xlsx): Over-represented GOs in genes negatively correlated with *ACE2* gene expression**

**Supplementary Table 8 (tableS8.docx): Pearson correlations and associated p-values of genes from the *TMPRSS* family with *ACE2* in the dataset GSE150819 containing lung organoids infected with SARS-CoV-2.**
